# The protein kinase G orthologs, EGL-4 and PKG-2, mediate serotonin-induced paralysis of *C. elegans*

**DOI:** 10.17912/micropub.biology.000115

**Published:** 2019-05-24

**Authors:** Andrew C. Olson, Michael R. Koelle

**Affiliations:** 1 Department of Molecular Biophysics and Biochemistry, Yale University School of Medicine, SHM CE30, New Haven, CT 06520-8024

**Figure 1:**
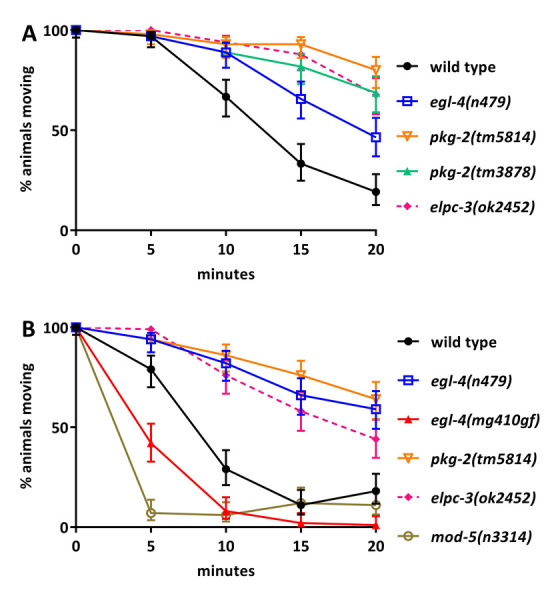
Time course of paralysis induced by treatment with serotonin in wild-type and mutant strains of *C. elegans*. Animals were placed in M9 liquid medium containing 10 mM serotonin and the animals still moving were counted at the indicated times. 100 animals were tested for each genotype. Error bars, 95% confidence intervals. Panel A and B assays were carried out on separate days. Some genotypes are shown in both panels to demonstrate the reproducibility of the assay. The *elpc-3(ok2452)* and *mod-5(n3314)* mutants are control strains previously shown (Gürel *et al.* 2012) to be serotonin resistant (*elpc-3*) or serotonin hypersensitive (*mod-5*). A) Loss-of-function mutants of *egl-4(n479)*, *pkg-2(tm3878),* and *pkg-2(tm5814)* are resistant to serotonin-induced paralysis; B) the *egl-4(mg410)* gain-of-function (gf) mutant is hypersensitive to serotonin-induced paralysis.

## Description

The *C. elegans* protein kinase G ortholog, EGL-4, has been demonstrated to be involved in *C. elegans* egg laying and the initiation of dwelling, a behavioral state characterized in part by low rates of locomotion (Trent *et al.* 1983; Hao *et al.* 2011). Serotonin regulates *C. elegans* egg laying through all five identified serotonin receptors, and serotonin promotes the dwelling state through the *mod-1* serotonin receptor (Trent *et al.* 1983; Hapiak *et al.* 2009; Flavell *et al.* 2013; Brewer *et al.* 2019). Given that serotonin plays a role in both the egg laying and dwelling behaviors that *egl-4* regulates, we sought to determine if protein kinase G is required for *C. elegans* to respond to serotonin. Exogenous serotonin paralyzes wild-type *C. elegans* (Gürel *et al.* 2012). The assays shown in Figure 1A indicate that one loss-of-function mutant of *egl-4* and two independent loss-of-function mutants of the *egl-4* paralog, *pkg-2* are resistant to paralysis by serotonin, similar to the previously-known serotonin resistant mutant *elpc-3(ok2452)* (Gürel *et al.* 2012)*.*The *pkg-2*(*tm3878)*and *pkg-2(tm5814)* alleles each carry deletions of sequences coding large portions of the conserved catalytic cGMP-dependent protein kinase domain of the PKG-2 protein, including the active site and ATP binding site (Hofmann *et al.* 1992), and thus we predict them to be null alleles. The *egl-4(n479)*mutation is an early stop codon prior to the kinase domain and *n479* is thus also predicted to be a null allele (Fujiwara *et al.* 2002). The Mak lab isolated an *egl-4* gain-of-function allele *mg410* that gives rise to a K162N single amino acid substitution (Hao *et al.* 2011). This K162N mutation lies in the conserved pseudo-substrate motif of EGL-4, and is predicted to result in auto-phosphorylation even in the absence of cGMP, leading to constitutively active EGL-4 (Hao *et al.* 2011). Figure 1B demonstrates that this *egl-4* gain-of-function mutant is hypersensitive to paralysis by serotonin, like the previously-known serotonin hypersensitive mutant *mod-5(n3314)* (Gürel *et al.* 2012), and in contrast to the serotonin-resistant *egl-4* and *pkg-2* loss-of-function mutants. Taken together these data indicate that the protein kinase G, *egl-4*, and its paralog *pkg-2* mediate serotonin-induced paralysis of *C. elegans*.

How do the protein kinase G orthologs, EGL-4 and PKG-2, mediate serotonin signaling in *C. elegans*? Previously our lab performed a forward genetic screen to identify proteins involved in serotonin signaling (Gürel *et al.* 2012). The proteins identified includedtwo serotonin receptors, SER-4 and MOD-1, and several of the other proteins were predicted to act in the SER-4 or MOD-1 pathways. SER-4 is a G protein coupled receptor (Olde and Mccombie 1997) and MOD-1 is a serotonin-gated chloride channel (Ranganathan *et al.* 2000). It is possible that protein kinase G is acting in the SER-4 or MOD-1 pathways to control the effects of serotonin on *C. elegans* locomotion. Alternatively, protein kinase G could act with the MOD-5 serotonin transporter (SERT). Prior work indicates that phosphorylation of mammalian SERT increases its activity and that protein kinase G acts in a pathway to stimulate SERT, although protein kinase G may not directly phosphorylate SERT (Miller and Hoffman 1994; Kilic *et al.* 2003; Ramamoorthy *et al.* 2007; Wong *et al.* 2012; Zhang *et al.* 2016). However, additional studies indicate that stimulation of cGMP pathways reduces SERT activity in certain cell types (Pogun *et al.* 1994; Asano *et al.* 1997). It is possible that *C. elegans* protein kinase G negatively regulates MOD-5 function.

## Methods

Assays were performed by filling microtiter wells with 50 µl of M9 buffer (Sulston and Hodgkin 1988). 10 animals were picked to each well. Prior to addition of serotonin, the number of animals moving in each well was counted to generate the zero time point (in all cases 100% of the population was moving at time zero). 50 µl of 20 mM serotonin in M9 buffer was then added to each well, so that assays were carried out at a final concentration of 10 mM serotonin. To dissolve serotonin in M9 buffer, the M9 buffer was first heated in a water bath to 90°C prior to the addition of 5-hydroxytryptamine creatine sulfate. The serotonin solution was then allowed to return to room temperature before use in assays. Wells were scored under a dissecting microscope for the number of moving animals at 5, 10, 15 and 20 minutes after the addition of serotonin. “Moving” was defined as having smooth swimming movements of the entire body. Animals showing only movements of the head or only stiff or jerky movements of ≤50% of the body were scored as not “moving.” 100 animals total were assayed for each genotype. Error bars are 95% confidence intervals calculated in Prism v.7.01 as part of a contingency table analysis using the Wilson/Brown method.

The *elpc-3(ok2452)* and *mod-5(n3314)* alleles were included as controls; they were previously shown to be respectively resistant and hypersensitive to paralysis by exogenous serotonin (Gürel *et al.* 2012). In this assay it is critical to test any two strains to be compared in parallel, as results vary somewhat for the same strain assayed from day to day. For example, in Figure 1A, at 15 minutes 33% of the wild-type population is still moving, while in Figure 1B, only 11% of the wild-type population is still moving at the same time point. Despite this experiment-to-experiment variability, certain mutants are always more resistant or more sensitive to serotonin than is the wild type. For example, the *egl-4* loss-of-function mutant is always more resistant to paralysis than is the wild type. In practice this means one can compare different strains within a single experiment, but cannot compare results from one experiment to the next.

## Reagents

5-hydroxytryptamine creatine sulfate complex; Sigma H-7752

The wild-type strain was Bristol N2. Strains available from *Caenorhabditis* Genetics Center are N2, MT1074 *egl-4(n479)* IV, and MT9772 *mod-5(n3314)* I. Strains available upon request are FX3878 *pkg-2(tm3878)* IV, FX5814 *pkg-2(tm5814)* IV, LX1769 *elpc-3(ok2452)* V, and VS39 *egl-4(mg410)* IV.

*tm3878* is a 313 bp deletion beginning with TAGGTTTTTATCTAGGACTA and ending with ATGGATGGCCAAAGCTCGTC.

*tm5814* is a 498 bp deletion beginning with GAAAAATTTCAAGTTTTAAG and ending with TGGCCAAGGTAAGATCCTCG.
